# Microwave ablation versus radiofrequency ablation for patients with primary and secondary hyperparathyroidism: a meta-analysis

**DOI:** 10.1007/s11255-023-03543-y

**Published:** 2023-03-09

**Authors:** Wei Xu, Shihui Li, Fang Cheng, Lifeng Gong, Weigang Tang, Jingkui Lu, Yani Li, Zhixia Wang

**Affiliations:** 1 Department of Nephrology, Wujin Hospital Affiliated with Jiangsu University, No. 2 Yongning Road, Changzhou, 213000 Jiangsu China; 2 Department of Nephrology, The Wujin Clinical College of Xuzhou Medical University, No. 2 Yongning Road, Changzhou, 213000 Jiangsu China; 3 Department of Laboratory Medicine, Jiangsu University, No. 301, Yongning Road, Xuefu Raod, Zhengjiang, 212013 Jiangsu China

**Keywords:** Hyperparathyroidism, Microwave ablation, Radiofrequency ablation, Meta-analysis

## Abstract

**Objective:**

Thermal ablation, including microwave ablation (MWA) and radiofrequency ablation (RFA), has been recommended for the treatment of primary hyperparathyroidism (PHPT) and refractory secondary hyperparathyroidism (SHPT). This meta-analysis was conducted to evaluate the efficacy and safety of MWA and RFA in patients with PHPT and refractory SHPT.

**Methods:**

Databases including PubMed, EMbase, the Cochrane Library, CNKI (China National Knowledge Infrastructure), and Wanfang were searched from inception to December 5, 2022. Eligible studies comparing MWA and RFA for PHPT and refractory SHPT were included. Data were analyzed using Review Manager software, version 5.3.

**Results:**

Five studies were included in the meta-analysis. Two were retrospective cohort studies, and three were RCTs. Overall, 294 patients were included in the MWA group, and 194 patients were included in the RFA group. Compared with RFA for refractory SHPT, MWA had a shorter operation time for a single lesion (*P* < 0.01) and a higher complete ablation rate for a single lesion ≥ 15 mm (*P* < 0.01) but did not show a difference in the complete ablation rate for a single lesion < 15 mm (*P* > 0.05). There were no significant differences between MWA and RFA for refractory SHPT concerning parathyroid hormone (*P* > 0.05), calcium (*P* > 0.05), and phosphorus levels (*P* > 0.05) within 12 months after ablation, except that calcium (*P* < 0.01) and phosphorus levels (*P* = 0.02) in the RFA group were lower than those in the MWA group at one month after ablation. There was no significant difference between MWA and RFA concerning the cure rate of PHPT (*P* > 0.05). There were no significant differences between MWA and RFA for PHPT and refractory SHPT concerning the complications of hoarseness (*P* > 0.05) and hypocalcaemia (*P* > 0.05).

**Conclusion:**

MWA had a shorter operation time for single lesions and a higher complete ablation rate for large lesions in patients with refractory SHPT. However, there was no significant difference in efficacy and safety between MWA and RFA in cases of both PHPT and refractory SHPT. Both MWA and RFA are effective treatment methods for PHPT and refractory SHPT.

## Introduction

Primary hyperparathyroidism (PHPT) is a common endocrine disease that ranks third most common after diabetes and thyroid disease and is caused by lesions of the parathyroid gland itself [1, 2]. Secondary hyperparathyroidism (SHPT) is a common and serious complication of end-stage renal disease caused by hypocalcaemia, hyperphosphataemia, and vitamin D deficiency [3–5]. The pathogenesis and clinical manifestations of PHPT and SHPT are different, but both PHPT and SHPT are characterized by elevated levels of parathyroid hormone (PTH) and bone mineral metabolism disorder. Severe hyperparathyroidism is associated with an increased risk of cardiovascular mortality [6, 7]. Parathyroidectomy (PTX) is a radical treatment for PHPT [8]. For refractory SHPT, PTX is also recommended in practical guidelines [5]. However, many patients, especially those with poor cardiopulmonary function, cannot tolerate PTX because it is invasive and traumatic.

Recently, thermal ablation using ultrasound guidance, as a minimally invasive treatment, has been used for the treatment of PHPT and refractory SHPT [9, 10]. Thermal ablation, including microwave ablation (MWA), radiofrequency ablation (RFA), laser ablation, and high-intensity focused ultrasound, aims to achieve thermal necrosis of the parathyroid glands. MWA and RFA are more commonly used for the treatment of PHPT and refractory SHPT, but the efficacy and safety of MWA and RFA are uncertain. Therefore, this meta-analysis was conducted based on the published literature to evaluate the efficacy and safety of MWA and RFA using ultrasound guidance in patients with PHPT and refractory SHPT.

## Materials and methods

### Search strategy

We searched the PubMed, EMbase, Cochrane Library, CNKI (China National Knowledge Infrastructure), and Wanfang databases from inception to December 5, 2022. The combined text and MeSH terms included primary hyperparathyroidism, secondary hyperparathyroidism, microwave ablation, and radiofrequency ablation. In addition, the relevant references and cited papers were searched manually to identify additional studies meeting the inclusion criteria. There were no language restrictions.

### Inclusion and exclusion criteria

The inclusion criteria were: (1) randomized, controlled trials (RCTs), cohort or case‒control studies; (2) symptomatic PHPT or asymptomatic PHPT with one of the following: (a) serum calcium level > 0.25 mmol/L greater than the upper limit of normal, (b) dual emission X-ray absorptiometry-derived T score < −2.5 at any part of the bone or/and history of fracture, (c) creatinine clearance < 60 ml/min, and (d) nephrolithiasis or increased stone risk [11, 12]; (3) refractory SHPT with one of the following: (a) persistent PTH levels > 800 pg/ml, (b) persistent hypercalcaemia and hyperphosphataemia and poor response to medical therapy, (c) parathyroid gland hyperplasia diagnosed by ultrasound or radionuclide imaging [13]; (4) comparison of outcomes between MWA and RFA; and (5) outcomes including at least one of the following indicators: operation time of ablation, complete ablation rate, serum PTH, serum calcium, serum phosphorus, cure rate and complications. Complete ablation was defined as an ablation zone completely covering the parathyroid nodule. The cure rate was defined as serum calcium and PTH decreasing to normal values for more than 6 months after ablation.

The exclusion criteria were: (1) case series, comments, reviews; (2) patients who had undergone surgical treatment; (3) those with severe coagulation disorders; (4) those with severe cardiopulmonary dysfunction; and (6) studies with a lack of relevant outcome data.

### Data extraction and quality assessment

Data were extracted independently by two investigators using standard data extraction forms. In cases of disagreement, a third investigator was consulted. We extracted characteristics including first author, year of publication, location, study design, sample size, mean age, sex, follow-up period, power of MWA and RFA, and treatment outcomes. The Cochrane assessment tool was used to assess the quality of RCTs [14], whereas the Newcastle–Ottawa scale (NOS) was used to assess nonrandomized studies [15].

### Statistical analysis

This meta-analysis was performed using Review Manager software, version 5.3 (Cochrane Collaboration). We summarized treatment outcomes as odds ratios (ORs) for categorical variables and weighted mean differences for continuous variables with 95% confidence intervals (CIs). *P* < 0.05 was considered statistically significant. We used the *I*^2^ statistic to assess heterogeneity among studies. We considered *I*^2^ > 50% and *P* < 0.10 to indicate significant heterogeneity. Meta-analysis with insignificant heterogeneity was performed using the fixed-effects model. For meta-analyses with significant heterogeneity, a random-effects model was used. Publication bias was assessed using subgroup analysis or sensitivity analysis.

## Results

### Study selection and characteristics

**Fig. 1:**
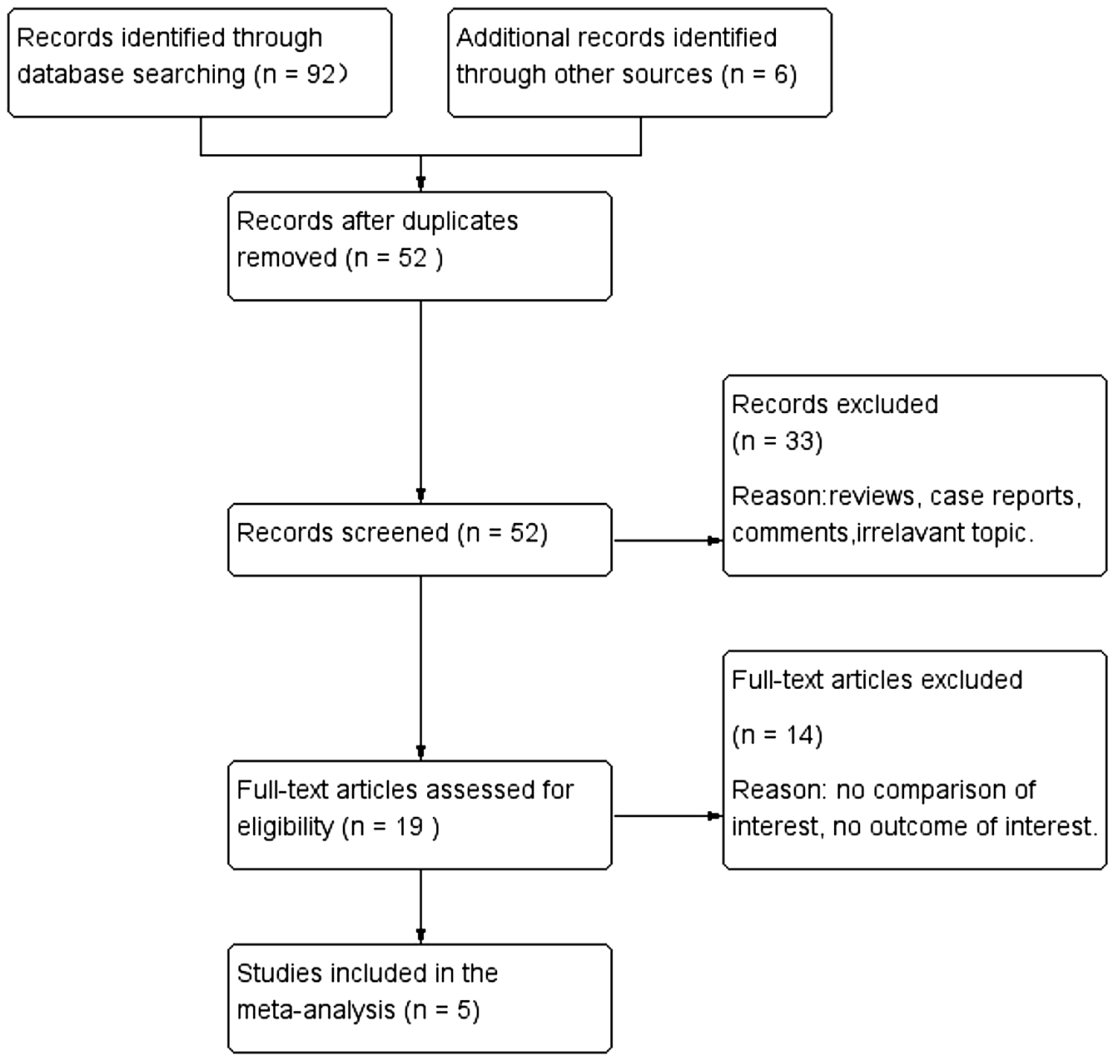
Flow diagram of the literature search

A flow diagram of the selection process is shown in Fig. 1. Finally, a total of five studies from China were included in this analysis [16–20]. Of the five studies, two were retrospective cohort studies, and three were RCTs. Overall, 294 patients were included in the MWA group, and 194 patients were included in the RFA group. The follow-up period was from 6 to 42.3 months. The risk of bias in the included RCTs was moderate. The cohort studies achieved scores of ≥ 6 points and were considered to be of high quality. The baseline characteristics of these studies are listed in Table 1. The Cochrane assessments are listed in Table 2, and the NOS assessments are listed in Table 3.

**Table 1:** Characteristics of the included studies

Study (year)	Country	Design	Follow-up period	Sample size	Mean age (years)	Male (*n* %)	PTH (pg/ml)	Calcium (mmol/L)	Phosphorus (mmol/L)	Power
Qinchun Pan 2021 [16]	China	RCT	12 months	MWA:30	50.68 ± 13.04	21 (70.0)	1650.73 ± 351.12	2.94 ± 0.52	2.83 ± 0.58	35 W
				RFA:30	49.56 ± 12.62	19 (63.3)	1635.62 ± 338.33	2.92 ± 0.54	2.81 ± 0.63	25 W
Rongrong Ru 2019 [17]	China	RCT	12 months	MWA:29	54.87 ± 10.94	20 (69.0)	1320.54 ± 472.13	2.96 ± 0.58	2.87 ± 0.76	–
				RFA:29	54.13 ± 11.98	18 (62.1)	1324.23 ± 456.78	2.89 ± 0.51	2.90 ± 0.50	25–35W
Jing Yuan 2021 [18]	China	RCT	6 months	MWA:68	41.51 ± 4.52	36 (52.9)	1320.29 ± 321.15	2.79 ± 0.30	2.93 ± 0.31	–
				RFA:67	40.54 ± 4.17	34 (50.7)	1325.52 ± 320.98	2.81 ± 0.28	2.91 ± 0.28	35W
Ying Wei 2021 [19]	China	Retrospective cohort study	9.6–42.3 months	MWA:77	55.5 ± 16.4	25 (32.5)	136.3	2.71 ± 0.23	0.86 ± 0.18	30 W or 35 W
				RFA:27	58.9 ± 15.6	9 (33.3)	157.2	2.76 ± 0.28	0.87 ± 0.15	
Fangyi Liu 2022 [20]	China	Prospective cohort study	6–36 months	MWA:91	57.33 ± 13.90	46 (34.9)	137.15	2.66	0.89 ± 0.19	–
				RFA:41						

*MWA* microwave ablation, *RFA* radiofrequency ablation

**Table 2:** Quality assessment of randomized control trial

Study	Random sequence generation	Allocation concealment	Blinding of participants and personnel	Incomplete outcome data	Selective reporting	Other bias
Qinchun Pan 2021 [16]	+	?	?	+	+	?
Rongrong Ru 2019 [17]	+	?	?	+	+	?
Jing Yuan 2021 [18]	+	?	?	+	+	?

The randomized control trial was evaluated using the Cochrane assessment tool

+  Low risk of bias

? Unclear risk of bias

- High risk of bias

**Table 3:** Quality assessment of cohort studies

Studies	Selection	Comparability	Outcome	Score
Ying Wei 2021 [19]	⋆⋆⋆	**⋆**	**⋆⋆**	6
Fangyi Liu 2022 [20]	**⋆⋆⋆⋆**	**⋆**	**⋆⋆**	7

The Cohort studies were evaluated using the Newcastle–Ottawa scale, which are comprised of the study of selection (representativeness of the exposed group, representativeness of the non exposed group, ascertainment of exposure, demonstration that outcome of interest was not present at start of study), group comparability (controls for the most important factor, controls for any additional factor),outcome measures (assessment of outcome, was follow-up long enough for outcomes to occur, adequacy of follow up of cohorts), a total of nine points

⋆ 1 point

### Meta-analysis results

#### The results of patients with refractory SHPT

##### Operation time of ablation

**Fig. 2:**
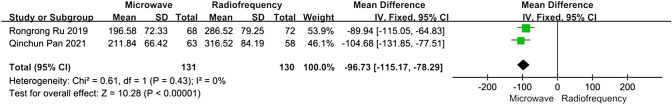
Forest plots comparing the operation time of single lesion between MWA and RFA group in patients with refractory SHPT

For patients with refractory SHPT, data about the operation time for ablation of a single lesion were reported in two articles. The heterogeneity between MWA and RFA was not substantial (*I*^2^ = 0%, *P* = 0.43). The operation time for a single lesion in the MWA group was significantly shorter than that in the RFA group (MD −96.73, 95% CI −115.17 to −78.29, *P* < 0.01) (Fig. 2).

##### Complete ablation rate for a single lesion

**Fig. 3:**
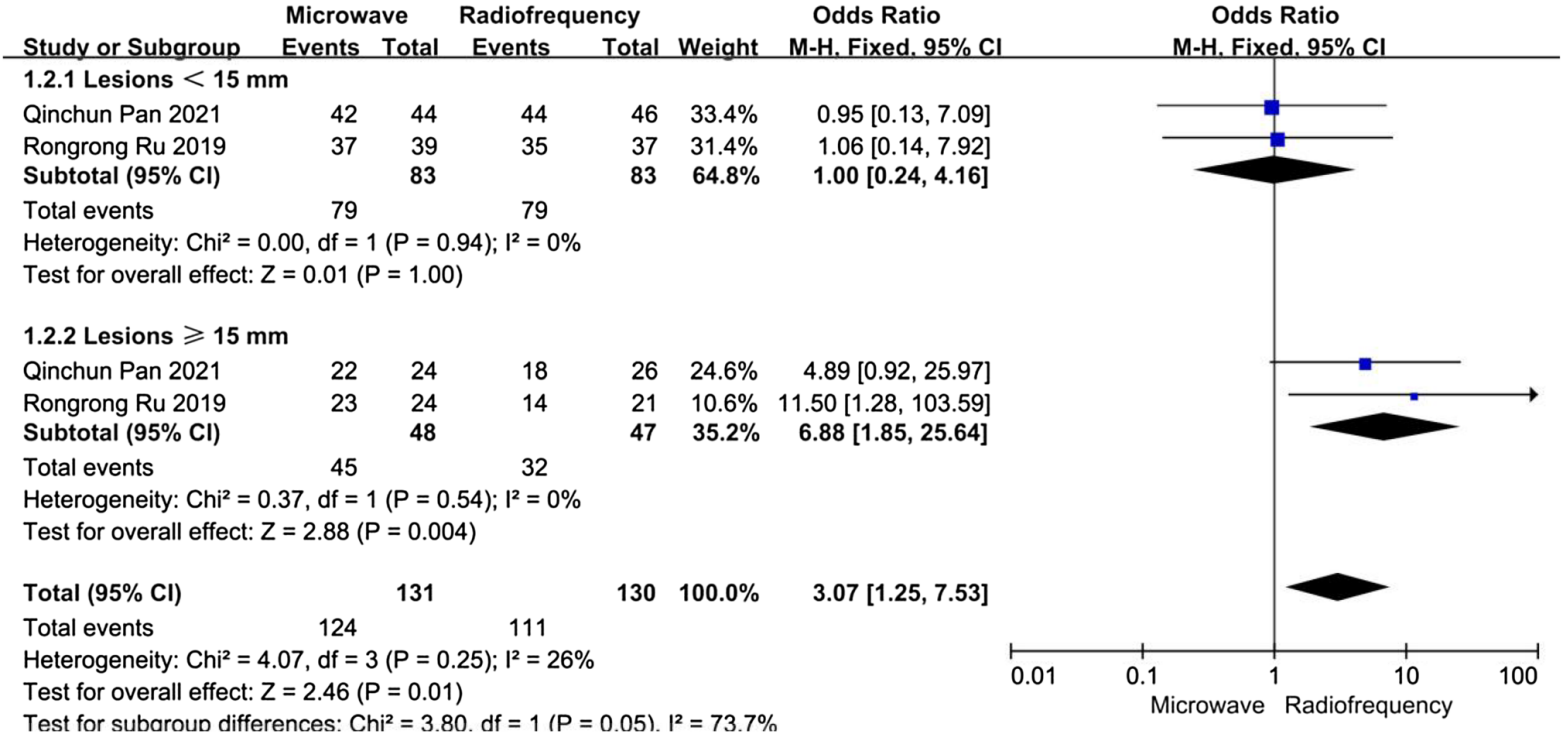
Forest plots comparing the complete ablation rate of single lesion between MWA and RFA group in patients with refractory SHPT

For patients with refractory SHPT, data about the complete ablation rate for a single lesion were reported in two articles. A subgroup analysis was performed according to whether the lesions were < 15 mm or ≥ 15 mm. In the lesions < 15 mm subgroup, the heterogeneity was not substantial (*I*^2^ = 0%, *P* = 0.94), and there was no significant difference between the MWA and RFA groups concerning the complete ablation rate for a single lesion (OR 1.00, 95% CI 0.24–4.16, *P* = 1.00). In the lesions ≥ 15 mm subgroup, the heterogeneity was not substantial (*I*^2^ = 0%, *P* = 0.54), and the complete ablation rate for a single lesion in the MWA group was higher than that in the RFA group; the difference was statistically significant (OR 6.88, 95% CI 1.85–25.64, *P* < 0.01) (Fig. 3).

##### PTH level

**Fig. 4:**
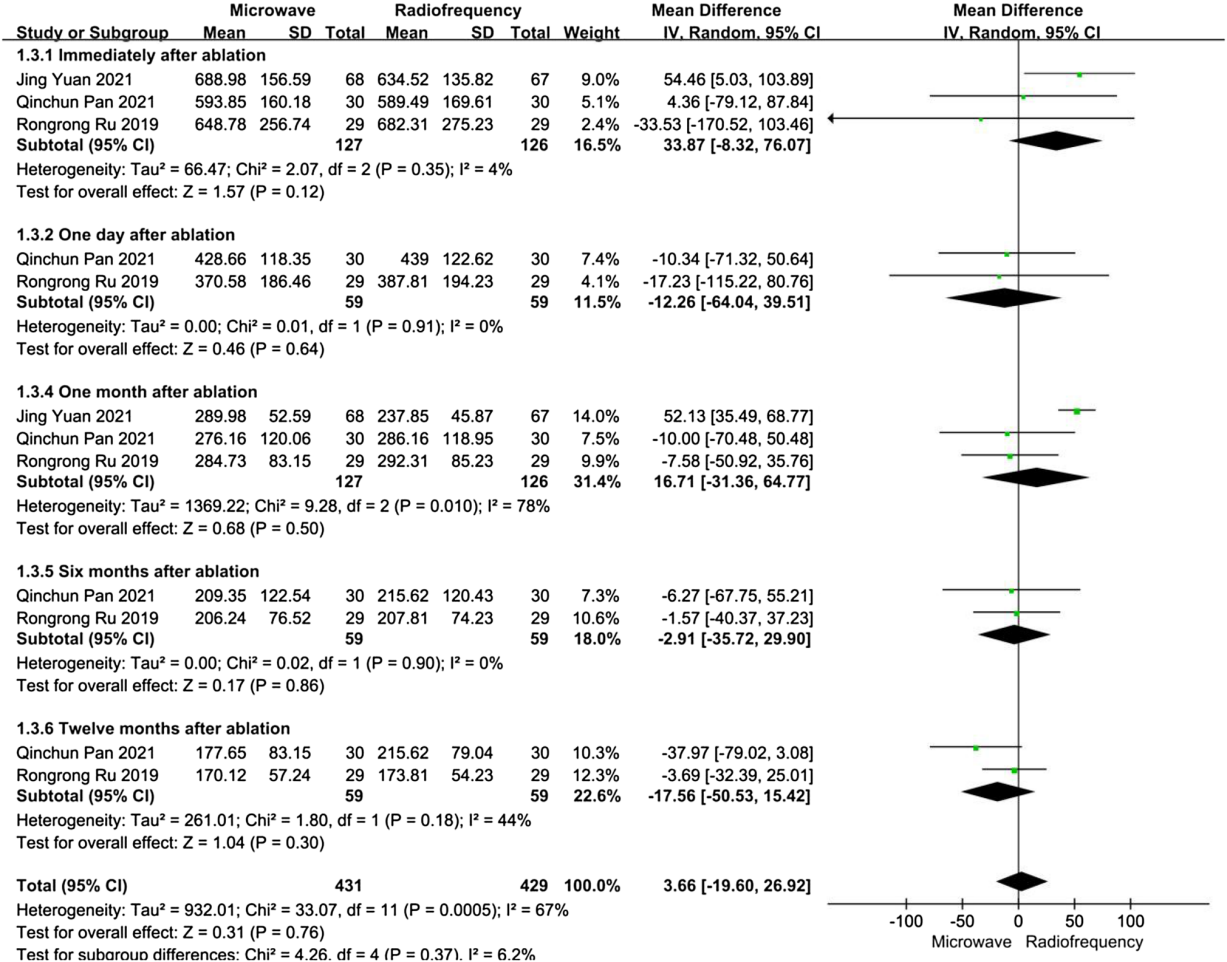
Forest plots comparing PTH levels between MWA and RFA group in patients with refractory SHPT

For patients with refractory SHPT, data about PTH levels after ablation were reported in three articles. We performed five subgroup analyses depending on the time after ablation. In the one month after ablation subgroup, the heterogeneity was substantial (*I*^2^ = 78%, *P* = 0.01). At other time points after ablation, the heterogeneities were not substantial (*I*^2^ < 50%, *P* > 0.10). In all subgroups, there was no significant differences between the MWA and RFA groups concerning PTH levels immediately, at one day, at one month, at six months, or at twelve months after ablation (*P* > 0.05) (Fig. 4).

We performed a sensitivity analysis. After Jing Yuan [18] was excluded from the one month after ablation subgroups, the heterogeneity was not substantial, and there was still no significant difference between the MWA and RFA groups concerning PTH levels.

##### Calcium level

**Fig. 5:**
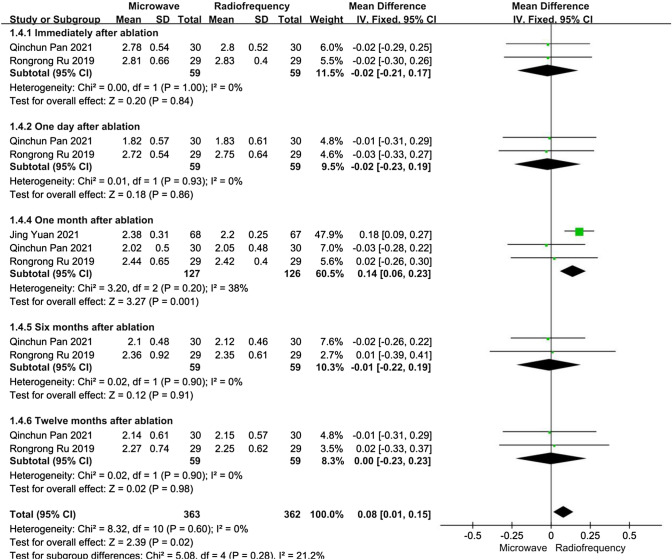
Forest plots comparing calcium levels between MWA and RFA group in patients with refractory SHPT

For patients with refractory SHPT, data about calcium levels after ablation were reported in three articles. We performed five subgroup analyses depending on the time after ablation. At all time points after ablation, the heterogeneities were not substantial (*I*^2^ < 50%, *P* > 0.10). One month after ablation, the calcium levels in the RFA group were lower than those in the MWA group, and the difference was statistically significant (MD 0.14, 95% CI 0.06–0.23, *P* < 0.01). However, except in the one month after ablation subgroup, there were no significant differences between the MWA and RFA groups concerning calcium levels immediately, at one day, at six months, or at twelve months after ablation (*P* > 0.05) (Fig. 5).

We performed a sensitivity analysis. After Jing Yuan [18] was excluded from the one month after ablation subgroups, there was no significant difference between the MWA and RFA groups concerning calcium levels at one month after ablation, without significant heterogeneity.

##### Phosphorus level

**Fig. 6:**
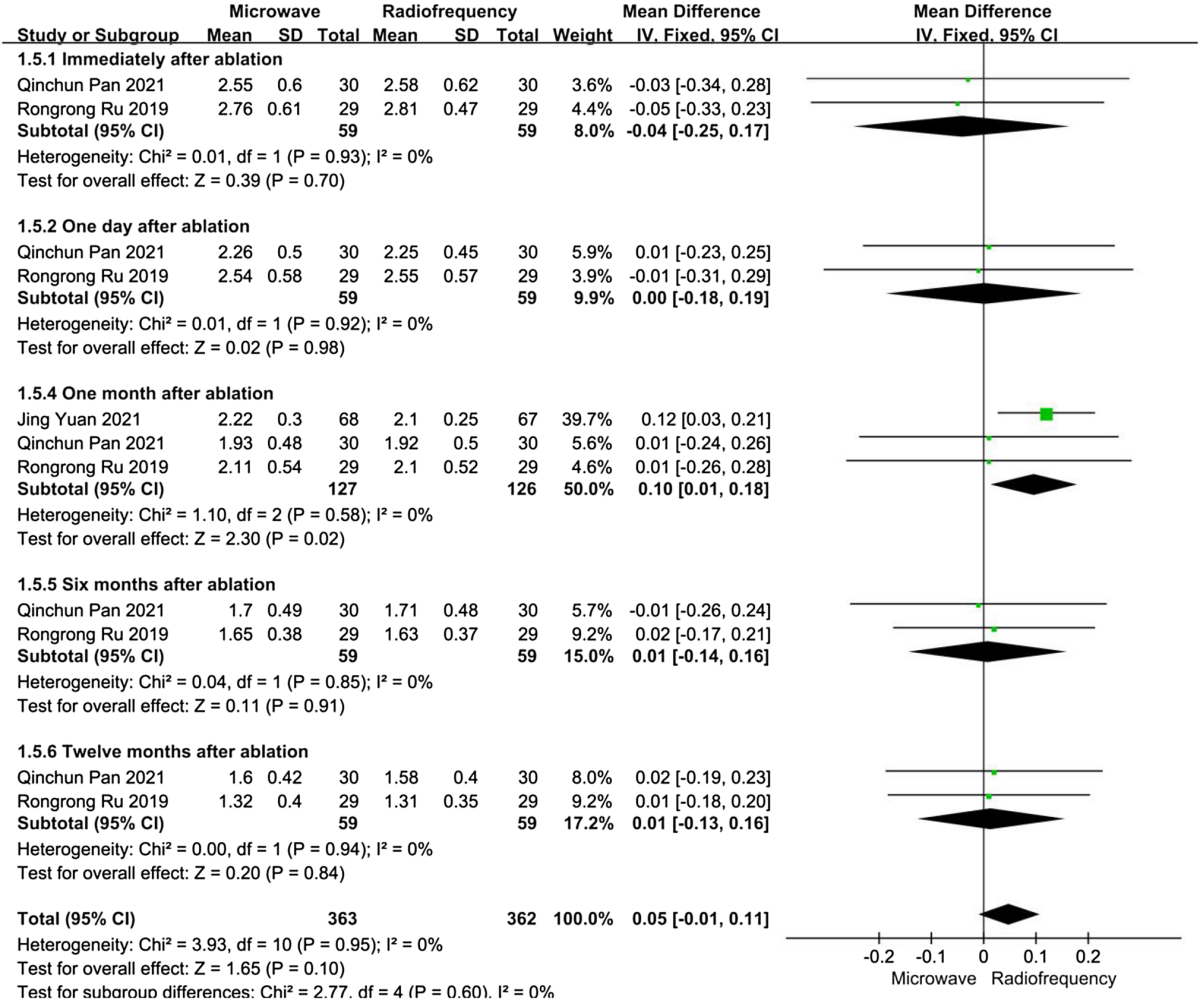
Forest plots comparing phosphorus levels between MWA and RFA group in patients with refractory SHPT

For patients with refractory SHPT, data about phosphorus levels after ablation were reported in three articles. We performed five subgroup analyses depending on the time after ablation. At all time points after ablation, the heterogeneities were not substantial (*I*^2^ < 50%, *P* > 0.10). One month after ablation, phosphorus levels in the RFA group were lower than those in the MWA group, and the difference was statistically significant (MD 0.11, 95% CI 0.01–0.15, *P* = 0.02). However, except in the one month after ablation subgroup, there were no significant differences between the MWA and RFA groups concerning phosphorus levels immediately, at one day, at six months, or at twelve months after ablation (*P* > 0.05) (Fig. 6).

We performed a sensitivity analysis. After Jing Yuan [18] was excluded from the one month after ablation subgroups, there were no significant differences between the MWA and RFA groups concerning phosphorus levels at one month after ablation, without significant heterogeneity.

#### The results of patients with PHPT

##### Cure rate

**Fig. 7:**
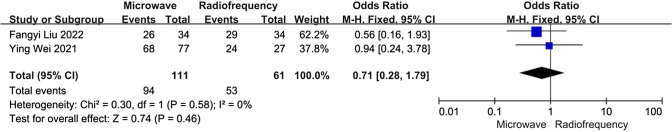
Forest plots comparing the cure rate between MWA and RFA group in patients with PHPT

For patients with PHPT, data about the cure rate after ablation were reported in two articles. The heterogeneity between the two studies was not significant (*I*^2^ = 0%, *P* = 0.58). There was no significant difference between the MWA and RFA groups concerning the cure rate after ablation (OR 0.71, 95% CI 0.28–1.79, *P* = 0.46) (Fig. 7).

#### Complications of patients with PHPT and refractory SHPT

##### Hoarseness

**Fig. 8:**
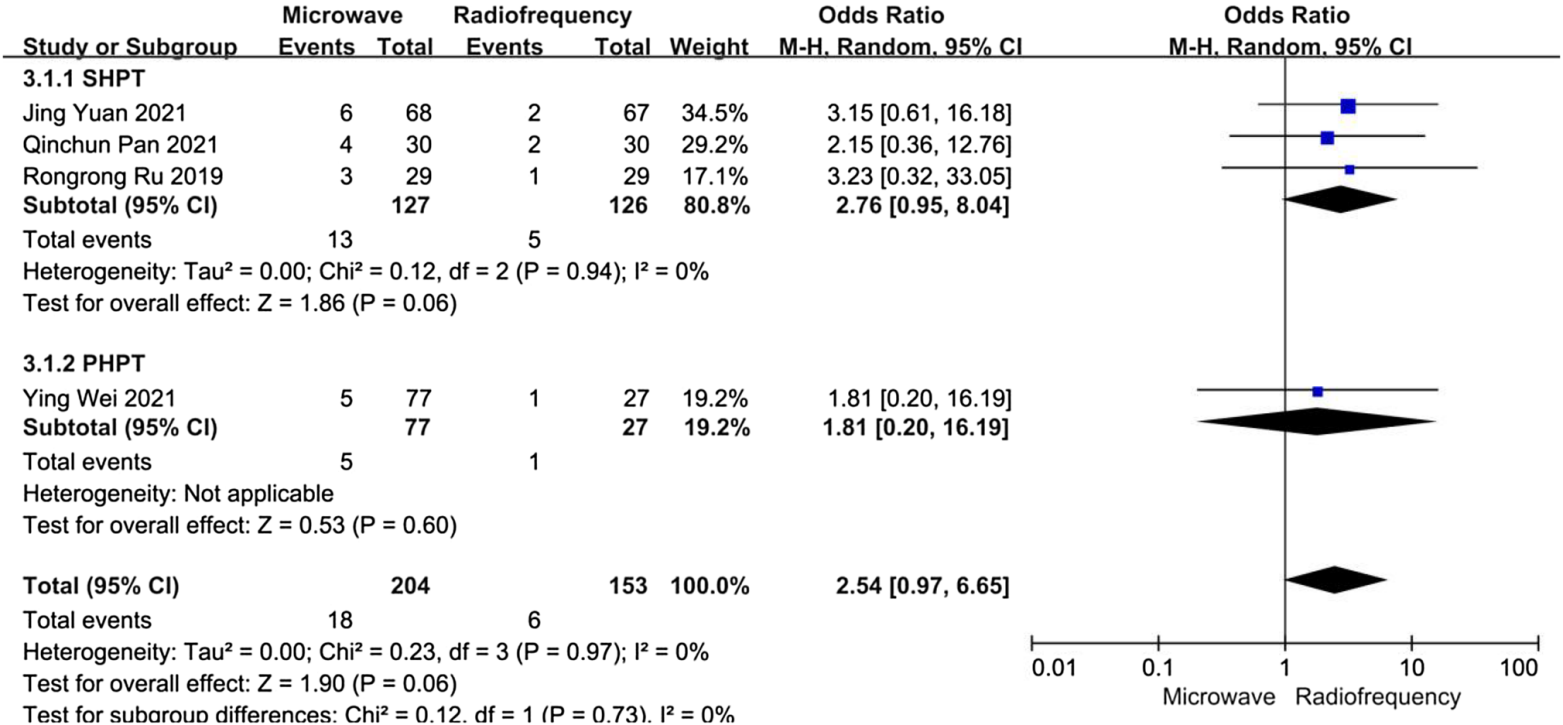
Forest plots comparing the incidence of hoarseness between MWA and RFA group in patients with PHPT and refractory SHPT

Data about the incidence of postoperative hoarseness were reported in four articles. A subgroup analysis was performed according to whether patients had PHPT or refractory SHPT. In the refractory SHPT subgroup, the heterogeneity was not substantial (*I*^2^ = 0%, *P* = 0.94), and there was no significant difference between the MWA and RFA groups concerning the incidence of hoarseness (OR 2.76, 95% CI 0.95–8.04, *P* = 0.06). In the PHPT subgroup, there was still no significant difference between the MWA and RFA groups concerning the incidence of hoarseness (OR 1.81, 95% CI 0.20–16.19, *P* = 0.60) (Fig. 8).

##### Hypocalcaemia

**Fig. 9:**
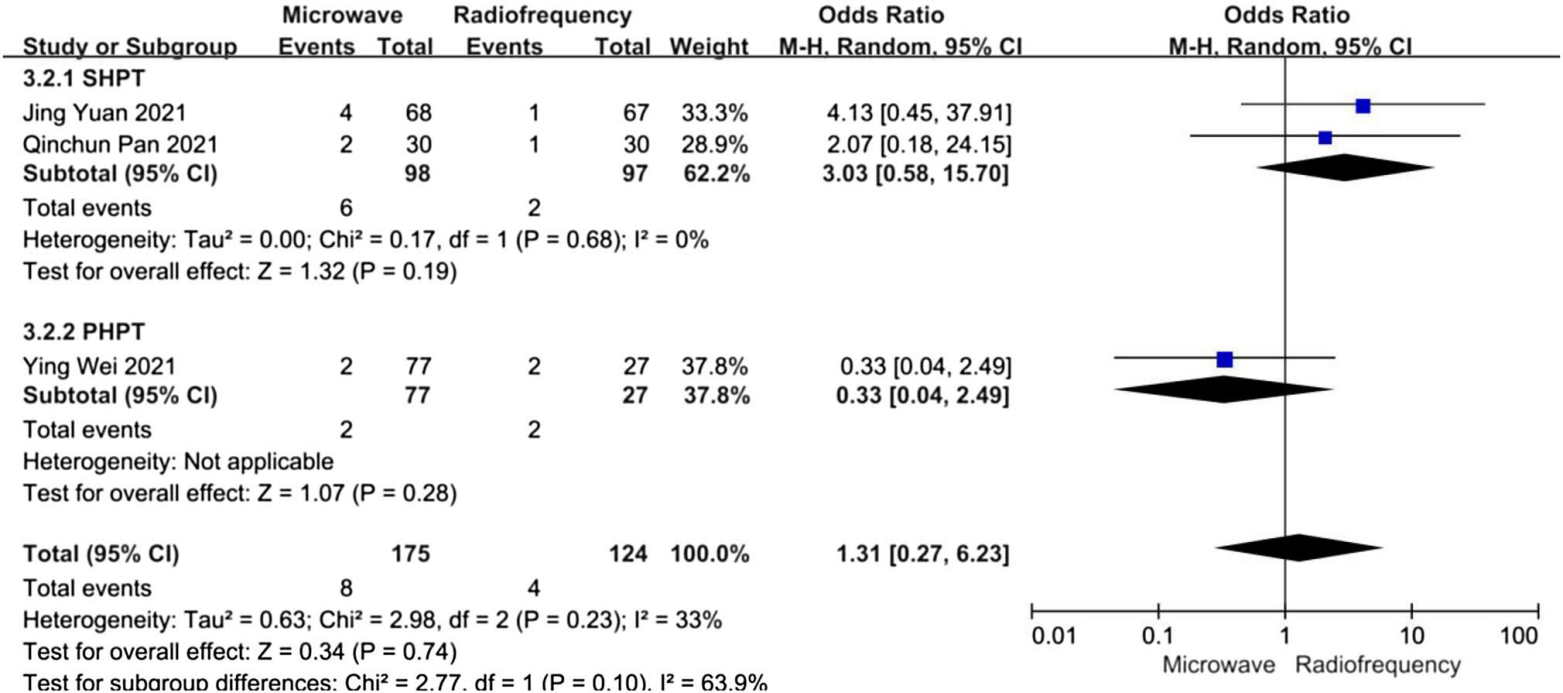
Forest plots comparing the incidence of hypocalcaemia between MWA and RFA group in patients with PHPT and refractory SHPT

Data about the incidence of postoperative hypocalcaemia were reported in three articles. A subgroup analysis was performed according to whether patients had PHPT or refractory SHPT. In the refractory SHPT subgroup, the heterogeneity was not substantial (*I*^2^ = 0%, *P* = 0.68,), and there was no significant difference between the MWA and RFA groups concerning the incidence of hypocalcaemia (OR 3.03, 95% CI 0.58–15.70, *P* = 0.19). In the PHPT subgroup, there was still no significant difference between the MWA and RFA groups concerning the incidence of hypocalcaemia (OR 0.33, 95% CI 0.04–2.49, *P* = 0.28) (Fig. 9).

## Discussion

Thermal ablation techniques, mainly MWA and RFA, aim to achieve thermal necrosis of parathyroid glands and have gradually shown obvious advantages in the treatment of PHPT or refractory SHPT [10]. Compared with PTX, thermal ablation has the advantages of minimal invasiveness, easy operation, fast recovery, and repeatable use for the treatment of PHPT or refractory SHPT [21]. However, there is no consensus regarding which is better in MWA and RFA concerning efficacy and safety for the treatment of PHPT or refractory SHPT.

We performed a meta-analysis in patients with refractory SHPT. We found that MWA had a shorter operation time for a single lesion than RFA, while compared with RFA, MWA had a higher complete ablation rate for a single lesion ≥ 15 mm, but MWA did not show an advantage in the complete ablation rate for a single lesion < 15 mm. This outcome can be explained by the differences in thermal efficiency and thermal energy density. The radiofrequency current operates only in very limited areas around the tip of the ablation needle, and the heat is conducted and diffused in a passive manner [22]. However, the range of microwave radiation is large, and all tissues in the path of radiation can generate heat at the same time, constituting an active process [23]. MWA has higher thermal efficiency and thermal energy density, which explains why the operation time was shorter, and the complete ablation rate of large lesions was higher in the MWA group.

MWA had a shorter operation time for a single lesion and a higher complete ablation rate for a large lesion than RFA, but we found that there were no significant differences in the efficacy concerning controlling PTH, calcium and phosphorus levels between MWA and RFA. It should be noted that, for patients with refractory SHPT, the calcium and phosphorus levels in the RFA group were lower than those in the MWA group at one month after ablation. After excluding the study of Jing Yuan [18], the sensitivity analysis showed that there were no significant differences between the MWA and RFA groups concerning calcium and phosphorus levels at one month after ablation. Due to the small number of included studies, we require more large RCTs to further compare the efficacy between MWA and RFA for patients with refractory SHPT. For patients with PHPT, there was still no significant difference between the MWA and RFA groups concerning the cure rate after ablation. In addition, for both primary and secondary hyperparathyroidism patients, it was certain that the levels of PTH, calcium and phosphorus after both types of ablation were closer to the normal level compared with those before ablation.

In terms of safety, we found that there was no significant difference between MWA and RFA. The main complications after MWA or RFA were hoarseness and hypocalcaemia in the included studies. Our meta-analysis revealed that MWA and RFA had similar incidences of hoarseness (8.8% versus 4.0%) or hypocalcaemia (4.6% versus 3.2%). Regarding hoarseness, there are three reasons, all of which are difficult to completely avoid in MWA and RFA procedures. The first reason of hoarseness is the temporary compression of the recurrent laryngeal nerve caused by the isolation fluid during the establishment of the parathyroid isolation zone [16, 17]. The second reason is the transient blocking of lidocaine [16]. The third reason is heat damage to the recurrent laryngeal nerve during the ablation process [17]. The patients with hoarseness can achieve spontaneous remission or be relieved by medications and physiotherapy. Regarding hypocalcaemia, the reason is the decrease in PTH levels after MWA or RFA [24, 25]. Our meta-analysis showed there was no significant difference concerning the PTH levels after MWA and RFA, which could explain why there was no difference in the incidence of hypocalcemia after MWA and RFA. Hypocalcaemia can be relieved by calcium supplementation.

There were some limitations in our meta-analysis. The number of included studies in our meta-analysis was small. Sensitivity analysis showed that the dependability of some outcomes was not sufficient. After excluding the study of Jing Yuan [18], the heterogeneity was changed in the one month after ablation subgroups concerning PTH, and the results concerning calcium and phosphorus levels after ablation were changed in the one month after ablation subgroup.

## Conclusions

Our meta-analysis revealed that MWA had a shorter operation time for a single lesion and a higher complete ablation rate for large lesions in patients with refractory SHPT. In cases of both PHPT and refractory SHPT, the levels of PTH, calcium and phosphorus after both types of ablation were better at achieving the normal level compared with those before ablation. However, there was no significant difference in efficacy and safety between MWA and RFA. To further confirm this conclusion, more large RCTs comparing MWA and RFA for the treatment of PHPT and refractory SHPT are necessary.
